# Wrist-Worn Electrodermal Activity as a Novel Neurophysiological Biomarker of Autonomic Symptoms in Spatial Disorientation

**DOI:** 10.3389/fneur.2018.01056

**Published:** 2018-12-04

**Authors:** Atsushi Tamura, Tetsuya Iwamoto, Hirokazu Ozaki, Mikihiko Kimura, Yukiko Tsujimoto, Yoshiro Wada

**Affiliations:** ^1^Department of Otolaryngology-Head and Neck Surgery, National Defense Medical College, Tokorozawa, Japan; ^2^Aeromedical Laboratory, Japan Air Self-Defense Force, Sayama, Japan; ^3^Department of Otolaryngology-Head and Neck Surgery, Nara Medical University, Kashihara, Japan

**Keywords:** electrodermal activity, spatial disorientation, motion sickness, autonomic nervous system symptoms, pilot

## Abstract

**Background:** Spatial disorientation is one of the most frequent causes of aircraft accidents, and is thus a major problem affecting air safety. Although a number of studies have examined spatial disorientation, the precise physiological changes occurring as a direct result of spatial disorientation and motion sickness remain unclear. The present study sought to investigate electrodermal activity (EDA) and subjective autonomic symptoms during spatial disorientation training, and to develop an indicator of physiological changes for pilot candidates.

**Methods:** In the current study, we investigated changes in EDA measured using a wrist-worn device, and subjective autonomic nervous system symptoms during spatial disorientation training for pilot candidates. We then used the Graybiel diagnostic criteria to develop a novel physiological biomarker.

**Results:** We found that maximum EDA change and peak amplitude were significantly increased in participants with a Graybiel score of ≥3 points compared with those who scored < 2 points. Furthermore, for symptoms of cold sweating and saliva secretion (from the seven Graybiel diagnostic criteria), the maximum EDA change in participants with scores ≥1 point was significantly higher than that of participants scoring 0 points.

**Conclusion:** Our results indicate that EDA data measured with a wrist-worn device could provide a useful method for objective evaluation of the severity of spatial disorientation and motion sickness.

## Introduction

In daily life, our brain subconsciously generates a mental image of our physical body in relation to the surrounding space. This image involves inner spatial axes corresponding to direction, position, size, shape, distance, and motion, and this function occurs both at rest and during motion in relation to space (1, 2). This ability of the brain to reproduce the physical space around us is called spatial orientation. Spatial orientation results from the integration of multiple sensory inputs from the visual, vestibular, and somatosensory systems in the brain (3, 4).

Spatial orientation in flight is sometimes difficult to achieve because the various types of sensory stimuli (visual, vestibular, and somatosensory inputs) vary in magnitude, direction, and frequency. Any differences or discrepancies between visual, vestibular, and somatosensory inputs result in a “sensory mismatch” that can produce illusions and lead to spatial disorientation (1, 2). In addition, when deprived of vision (e.g., because of clouds, fog, or low-light conditions at night), even experienced aircraft pilots may be unable to maintain spatial orientation, and commonly experience spatial disorientation. Spatial disorientation can lead to several autonomic symptoms that are commonly referred to collectively as motion sickness, including nausea, salivation, and cold sweating (5, 6).

Numerous studies have evaluated spatial disorientation and motion sickness over many years. Objective evaluations of motion sickness using physiological indices have utilized various measures, including electroencephalographic responses (7), changes in heart rate, blood pressure, and body temperature (8), altered breathing and maximum oxygen intake (9), and characteristic eye movements (10). However, evaluating the onset mechanisms and physiological changes that occur during spatial disorientation remains difficult (8) because spatial disorientation affects the visual and vestibular systems, making it difficult to simultaneously evaluate all *in vivo* changes. In addition, even if various physiological or biochemical markers are used, individual differences exist in the degree of spatial disorientation, making it difficult to develop a method for objective evaluation. Furthermore, devices for measuring spatial disorientation must be sufficiently portable for measuring physiological markers during movement.

In recent years, various devices have been tested for spatial disorientation training in pilots (11–14). However, despite intensive efforts in the development of spatial disorientation training programs (11–14), advanced hardware (10–14), and research (7–10, 14), the operational impact of spatial disorientation in terms of crew and aircraft losses remains significant. Conventional spatial orientation training is primarily composed of lectures on the anatomy and physiology of the sensory systems. Significant efforts have also been made to reproduce various types of visual and vestibular spatial disorientation that pilots might encounter in flight, with limited and variable success.

Electrodermal activity (EDA) refers to the electrical changes measured at the surface of the skin that arise when the skin receives innervating signals from the brain, and can be used to evaluate sympathetic activity (15–17). For most people, emotional arousal, increased cognitive workload, and/or physical exertion cause the brain to send signals to the skin, thus increasing the level of sweating. Although this increase in sweat on the surface of the skin may not be noticeable, electrical conductance increases significantly as the pores of the skin begin to fill below the surface (18–20).

Past studies have investigated changes in autonomic nervous system symptoms using EDA as an indicator of spatial disorientation and motion sickness (14, 21, 22). Some previous studies have reported that EDA exhibits marked changes with the degree of spatial disorientation and motion sickness, and that EDA levels are sensitive to simulator sickness (14, 21). However, other studies have reported that EDA does not change significantly with these variables (22). Therefore, it remains unclear whether EDA changes during spatial disorientation and motion sickness.

In previous studies, EDA measurements were carried out by attaching electrodes to the palm, fingers, and ankle (18, 22). However, in addition to requiring several electrodes, these electrodes typically detach easily, making EDA difficult to measure accurately (18, 22).

Recently, a number of researchers have measured EDA using wrist-worn devices to specifically evaluate autonomic nerve function in patients with various diseases, and to monitor activity during sleep (16, 23–25). However, to the best of our knowledge, no previous studies have measured EDA using a wrist-worn device during spatial disorientation training. Thus, we hypothesized that EDA levels measured using a wrist-worn device may provide a novel neurophysiological biomarker that could be used to objectively evaluate the degree of spatial disorientation.

Using a different approach, Gordon et al. examined the components of saliva as an objective assessment of motion sickness, reporting that the amount of amylase in saliva was significantly higher in people who are prone to motion sickness compared with those who are not (26). In addition, the researchers suggested that the amount of amylase in saliva could provide an indicator of a person's susceptibility to motion sickness. They interpreted their results as indicating that the sympathetic nervous system tended to work more actively for people who were prone to motion sickness because amylase secretion in saliva is regulated by the sympathetic nervous system. Therefore, in the current study, we hypothesized that alterations in autonomic function would also be reflected in EDA changes, alongside salivary secretion and various autonomic neurological symptoms.

The present study sought to investigate EDA using a wrist-worn device while measuring subjective autonomic symptoms during spatial disorientation training, as well as examining the correlation between these variables. Overall, we sought to develop an indicator of physiological changes during spatial disorientation training for pilot candidates.

## Materials and Methods

### Participants

A group of 177 healthy pilot candidates aged 22–25 years old (mean age: 22.7 years; male/female: 170/7) participated in this study. All participants belonged to the Japan Air Self-Defense Force. Participants had no previous medical history of eye, ear, or equilibrium disorders. We carried out an interview with each participant about their physical and emotional condition before the experiment, and confirmed that no participants had abnormal health conditions, and none were sleepy, hungry, or thirsty before commencing spatial disorientation training. The protocol was approved by the Ethics Committee of the Aeromedical Laboratory (Notification No. 25-2-1). Written informed consent was obtained from each participant, and the investigations were conducted in accord with the principles of the Declaration of Helsinki.

### Device for Spatial Disorientation Training

**Figure 1:**
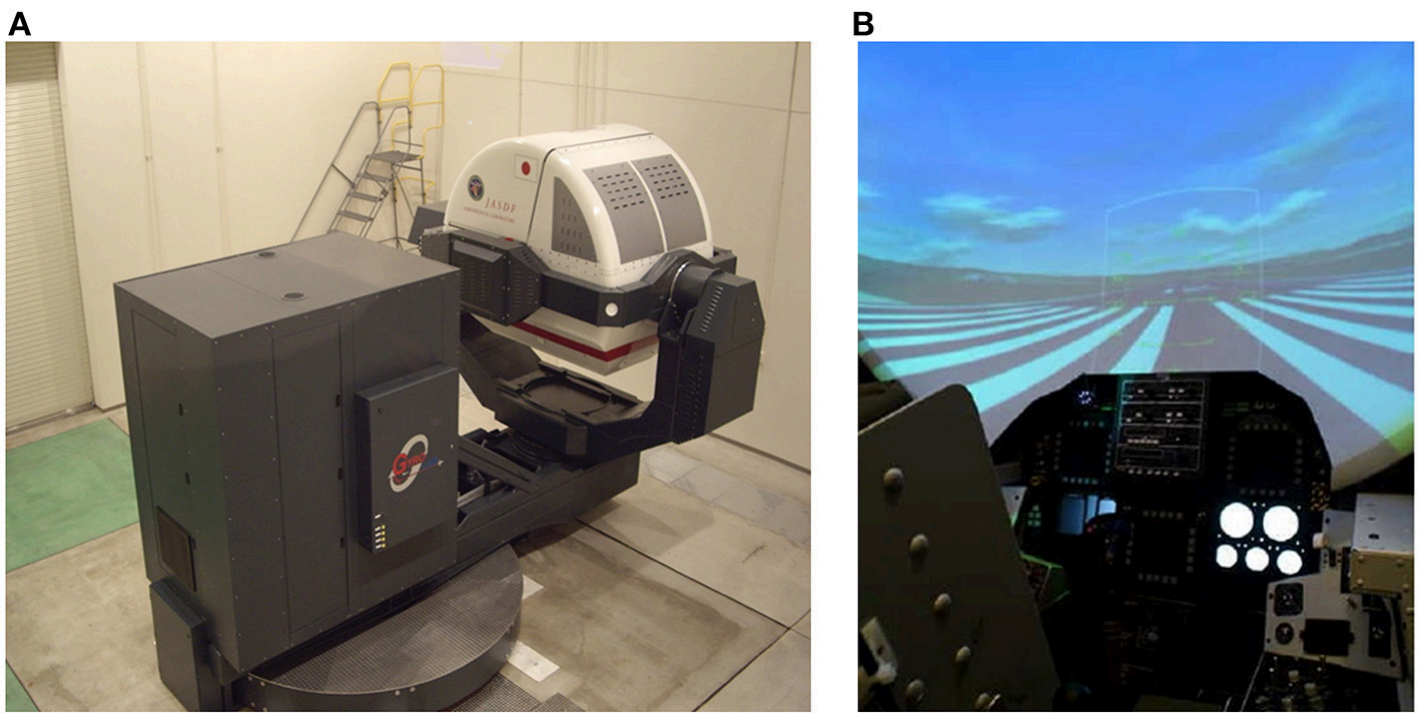
Device used to produce a variety of tilt environments with visual stimulation. Publication of these images has been approved by the Aeromedical Laboratory, Japan Air Self-Defense Force. **(A)** Spatial orientation training device, GYROLAB GL-4000. **(B)** Inside the cockpit. Images were projected onto a screen in front of the participants.

We used a spatial disorientation training device GYROLAB GL-4000 (Environmental Tectonics Corporation, Southampton, PA, USA) located at the Aeromedical Laboratory of the Japan Air-Self Defense Force to produce a variety of spatial disorientation environments (10). The cockpit of this training device had degrees of freedom on four axes: planetary (3.05-m radius), pitch, roll, and yaw (Figure 1A). In the cockpit, animated images and still images were presented on a screen (120 × 70° field of view) located 0.9 m in front of the participants using a projector (Figure 1B). We monitored participants' head position in real time through a charge-coupled device (CCD) camera mounted in the cockpit. During training, we communicated with participants using headsets. Participants were securely fastened into their seats via five-point seatbelts. Participants' heads were aligned with the headrest of the seat and were not fixed in place, enabling them to sit in a way that approximated the posture of an active pilot (10).

### Spatial Disorientation Training for Pilot Candidates

We used a spatial orientation training device to present visual and vestibular stimuli to participants as spatial disorientation training. In the daytime simulation (i.e., daytime takeoff, turning operation during daytime), we used the projector to present animated images consisting of a runway, towns, forests, mountains, clouds, and a blue sky. Nighttime simulation included the same images, but with poor visual information, and contained runway approach lights, town lights, and stars. In the nighttime simulation (i.e., nighttime takeoff, turning operation at night), we reduced brightness so that outlines (such as those of mountains or buildings) disappeared and spatial information was poor.

The content of the spatial disorientation training is shown in Table 1. False visual reference illusions may cause pilots to orient their aircraft in relation to a false horizon; these illusions can be caused by flying over a banked cloud, night flying over a featureless terrain with ground lights that are indistinguishable from a dark sky with stars, or night flying over a featureless terrain with a clearly defined pattern of ground lights and a dark, starless sky. The somatogravic illusion involves a sudden forward linear acceleration during level flight, in which the pilot experiences the illusory perception that the nose of the aircraft is pitching up. This illusion is caused by a sudden return to level flight following a gradual and prolonged turn that went unnoticed by the pilot. Similarly, the Coriolis illusion involves the simultaneous stimulation of two semi-circular canals, and is associated with a sudden tilting (forward or backwards) of the pilot's head while the aircraft is turning. This illusion can occur when a pilot tilts their head down (to look at an approach chart or to write a note on their knee board), up (to look at an overhead instrument or switch), or sideways. The graveyard spin is an illusion that pilots may experience when they intentionally or unintentionally enter a spin (Table 1).

**Table 1:** Spatial disorientation training for candidate pilots.

1	Daytime take off	Take off at daytime
2	Nighttime take off	Take off with poor visual information at nighttime
3	Turning operation at daytime	Daytime flight
4	False visual reference illusions	A false visual reference illusion can be caused by city lights, clouds, stars, and darkness.
5	Turning operation in clouds	Flight in environments with poor visual information in clouds
6	False visual reference illusions	A false visual reference illusion can be caused by city lights, clouds, stars, and darkness.
7	Turning operation at nighttime	Flight in environments with poor visual information at nighttime
8	False visual reference illusions at nighttime	A false visual reference illusion can be caused by city lights, clouds, stars, and darkness.
9	Somatogravic illusion	The rapid acceleration pushes the pilot back in their seat, giving them the sensation of a nose up attitude. To correct this, the pilot noses the plane over toward the earth.
10	The Leans	The leans occur when a quick correction of a banked attitude happens too slowly. The sensory membrane in the inner ear sends the brain information inducing the sensation of banking in the opposite direction. However, the disoriented pilot tends to over-bank in the wrong direction, potentially rolling the aircraft.
11	Loop	Loop inside and outside clouds in the daytime.
12	Coriolis illusion	The Coriolis illusion is caused by making a quick head movement during a constant rate turn that has ceased stimulating the inner ear.
13	Graveyard spin	When a pilot is recovering from a spin that has stopped, the fluid in the inner ear can create the illusion that they have entered a spin in the other direction.

*Participants were exposed to the visual illusion, vestibular illusion, and a combination of the two using a spatial disorientation training device*.

**Figure 2:**
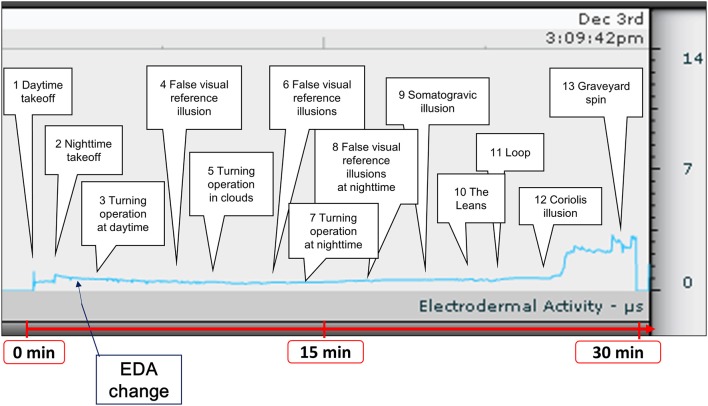
Time course of spatial disorientation training and a representative example of EDA measurement. Participants were exposed to spatial disorientation training, including a visual illusion, vestibular illusion, and a combination of the two for ~30 min. We recorded EDA changes during training. EDA, electrodermal activity.

We exposed participants to a visual illusion, vestibular illusion, or a combination of the two for 30 min using the spatial disorientation training device (Figure 2). Spatial disorientation training was conducted according to the protocols defined by the Japan Air Self-Defense Force, and the training time and EDA measurement time were the same for all participants.

### Evaluation of Electrodermal Activity

**Figure 3:**
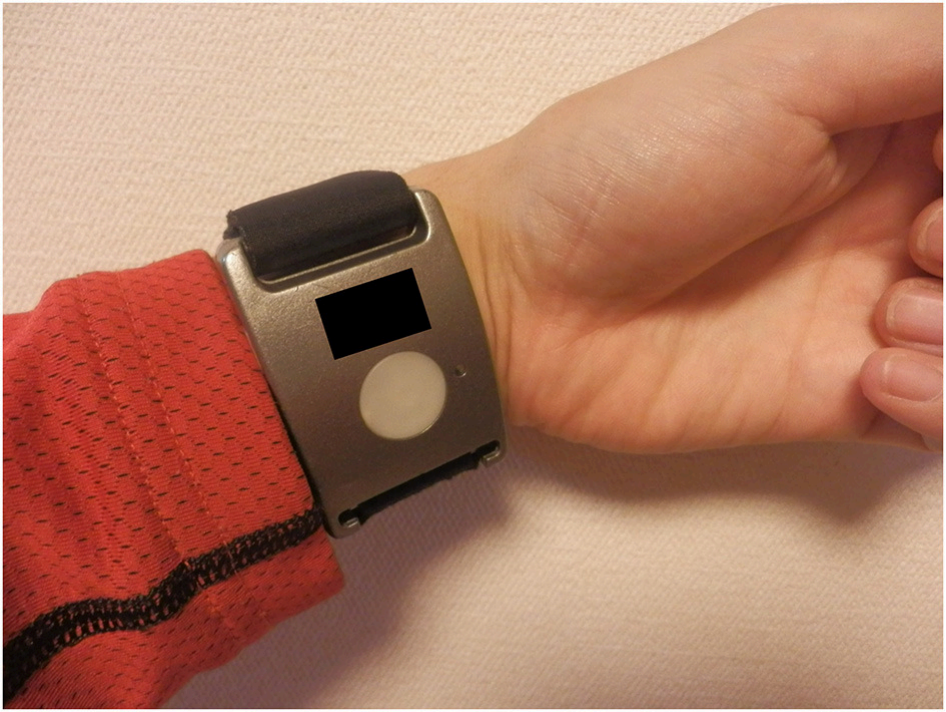
Device used to evaluate EDA. The wrist-worn EDA evaluation device, Q sensor. EDA, electrodermal activity.

To investigate the changes in EDA obtained using a wrist-worn device during spatial disorientation training, we used a device called the Q sensor 2.0 (Affectiva, Waltham, MA, USA) (Figure 3). The Q sensor measures electrical conductance (the inverse of resistance) across the skin, by passing a minuscule amount of direct current between two electrodes that are in contact with the skin. The units of measurement in this system are microsiemens (μS). Participants were asked to wear the Q sensor during spatial disorientation training. In this experiment, all participants wore the Q sensor on their left wrists because they were required to operate switches in the spatial orientation training device with their right hands, causing movement that could affect the skin conductance data. To avoid this problem, we fixed the left hand of each participant in position beside the seat. The position was confirmed via a CCD camera in the training device.

After training, we reviewed and annotated the EDA data recorded by the Q sensor. Two 12-mm disposable silver-coated electrodes were applied to the volar surface of the wrist on the side without arterial line access. The raw EDA signals were downloaded daily and the data were analyzed off-line using company-provided software (Affectiva Q version 2.01.56) (Figure 2). We then calculated the maximum change in EDA, the number of EDA peaks, the mean and median amplitude, and the area under the curve (AUC) values for the EDA data in all participants. We defined the maximum change as the difference between the minimum EDA value before spatial disorientation training and the maximum EDA value during training. Peak EDA measurements were visually evaluated using every short-term phasic increase in EDA >0.01 μS (27). We used ImageJ software version 1.44p (http://rsbweb.nih.gov/ij/download.html) to measure the AUC (28). The units of measurement were pixels.

### Evaluation of Subjective Autonomic Symptoms

To evaluate subjective autonomic symptoms, we used the Graybiel diagnostic score and quantified the severity of autonomic symptoms during spatial disorientation training (29) (Table 2). Two examiners observed and evaluated the complexion and sweating state of the participants, and scored the data after training, according to the diagnostic categorization (29) (Table 2).

**Table 2:** Evaluation of subjective autonomic symptoms.

**Category**	**Pathognomonic 16 points**	**Major 8 points**	**Minor 4 points**	**Minimal 2 points**	**AQS 1 point**
Nausea syndrome	Vomiting	Nausea 2, 3	Nausea 1	Epigastric discomfort	Epigastric awareness
Skin color		Pallor 3	Pallor 2	Pallor 1	Flushing
Cold sweating		3	2	1
Increased salivation		3	2	1
Drowsiness		3	2	1
Pain					Headache
Central nervous system					Dizziness

*We used the Graybiel diagnostic criteria and quantified the severity of autonomic symptoms. AQS, additional qualifying symptoms*.

We divided participants into three groups according to their total Graybiel scores: (1) 0 points (*n* = 59), (2) 1–2 points (*n* = 64), and (3) ≥3 points groups (*n* = 54). In addition, we compared the maximum EDA change between these three groups.

We also divided the participants into two groups according to Graybiel scores, for each autonomic symptom: (1) 0 points group and (2) ≥1 points group for the seven autonomic symptoms. For the nausea syndrome, we divided participants into: (1) 0 points group (*n* = 104) and (2) ≥1 points group (*n* = 73). For skin color, we divided participants into: (1) 0 points group (*n* = 122) and (2) ≥1 points group (*n* = 55). For cold sweating, we divided participants into: (1) 0 points group (*n* = 153) and (2) ≥1 points group (*n* = 24). For increased salivation, we divided participants into: (1) 0 points group (*n* = 151) and (2) ≥1 points group (*n* = 26). For drowsiness, we divided participants into: (1) 0 points group (*n* = 162) and (2) ≥1 points group (*n* = 15). For pain, we divided participants into: (1) 0 points group (*n* = 168) and (2) ≥1 points group (*n* = 9). For central nervous system function, we divided participants into: (1) 0 points group (*n* = 160) and (2) ≥1 points group (*n* = 17).

In addition, we compared the maximum EDA change between the two groups for each autonomic symptom.

### Statistical Analysis

We assessed the maximum EDA change, the number of EDA peaks, the mean EDA amplitude, and the AUC values for the EDA data among the three groups using one-way analysis of variance (ANOVA) with Tukey-Kramer multiple comparison tests. The relationship between the maximum EDA change and the total EDA score in the ≥3 points group was investigated using Pearson's correlation coefficients after detecting outliers using the Smirnov-Grubbs test. The maximum EDA change between the two groups for each autonomic symptom was assessed using Welch's *t*-tests, and Cohen's *d* was used to calculate effect sizes. A value of *p* < 0.05 was considered statistically significant.

## Results

### Evaluation of EDA and Subjective Autonomic Symptoms

**Figure 4:**
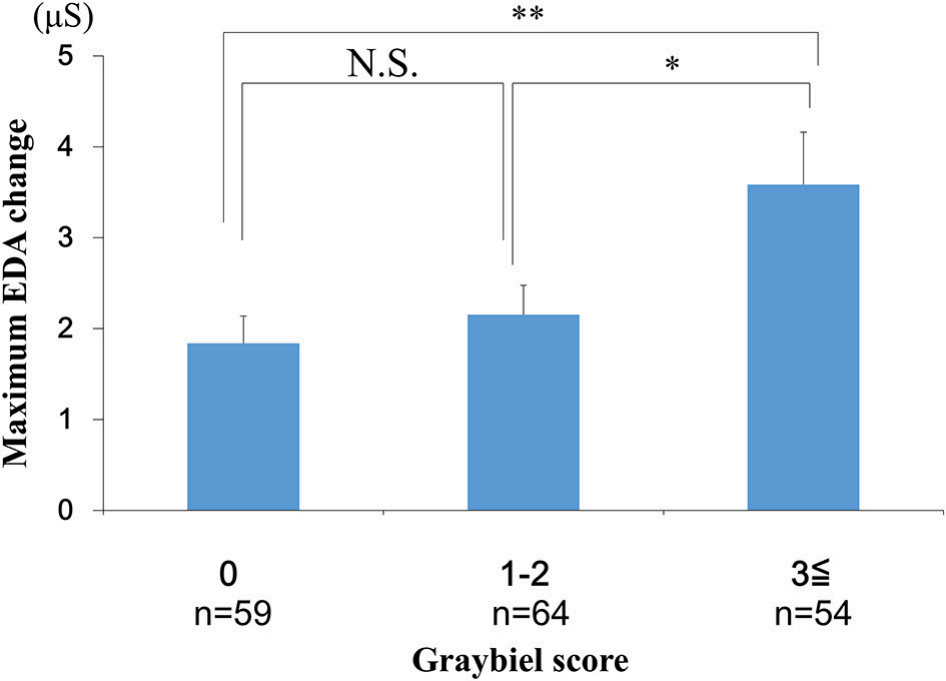
Evaluation of EDA and subjective autonomic symptoms during spatial disorientation training using the Q sensor and Graybiel criteria, respectively. The maximum amount of change was significantly greater in the ≥3 points group compared with the 0 points group (*p* < 0.01) and the 1–2 points group (*p* < 0.05). EDA, electrodermal activity. The error bars represent the mean (S.E.) in all graphs. ^*^p < 0.05, ^**^p < 0.01.

We conducted EDA analysis during and immediately after spatial disorientation training (Table 1), and administered a questionnaire about autonomic symptoms experienced during training. To measure autonomic symptoms, we used Graybiel's diagnostic criteria and scored participants accordingly (29) (Table 2). We were able to measure EDA in all participants during training (Figure 2 and Supplementary Table 1). The maximum amount of change in EDA was significantly higher in the ≥3 points group compared with the 0 points group (*p* < 0.01) and the 1–2 points group (*p* < 0.05) (Figure 4). We observed no significant differences between the 0 points and 1–2 points groups (Figure 4).

### Correlation Between Maximum EDA Change and Total Graybiel Score in the ≥3 Points Group

**Figure 5:**
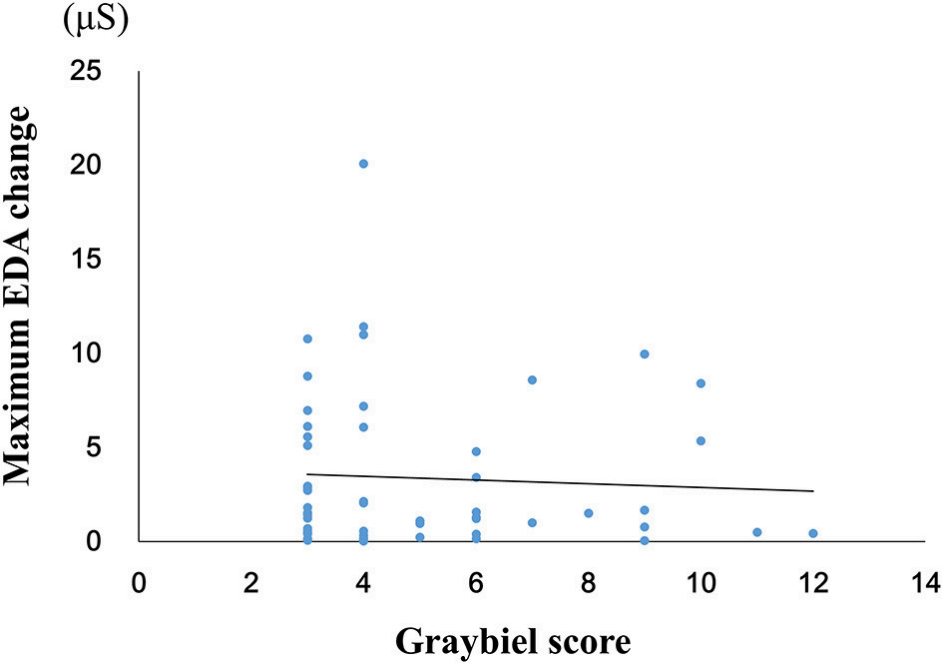
Relationship between the maximum amount of EDA change and the total Graybiel score in the ≥3 points group. No significant (*p* > 0.05) correlation (*r* = −0.059) was observed between the maximum EDA change and the total Graybiel score.

We investigated the relationship between the maximum EDA change and the total Graybiel score in the ≥3 points group (Figure 5). We identified and excluded two outliers (with scores of 18 points and 36 points) with respect to Graybiel scores using the Smirnov-Grubbs test. No significant (*p* > 0.05) correlation (*r* = −0.059) was observed between the maximum change in EDA and the total Graybiel score.

### Evaluation of the Number of EDA Peaks, the Mean EDA Amplitude and Subjective Autonomic Symptoms

**Figure 6:**
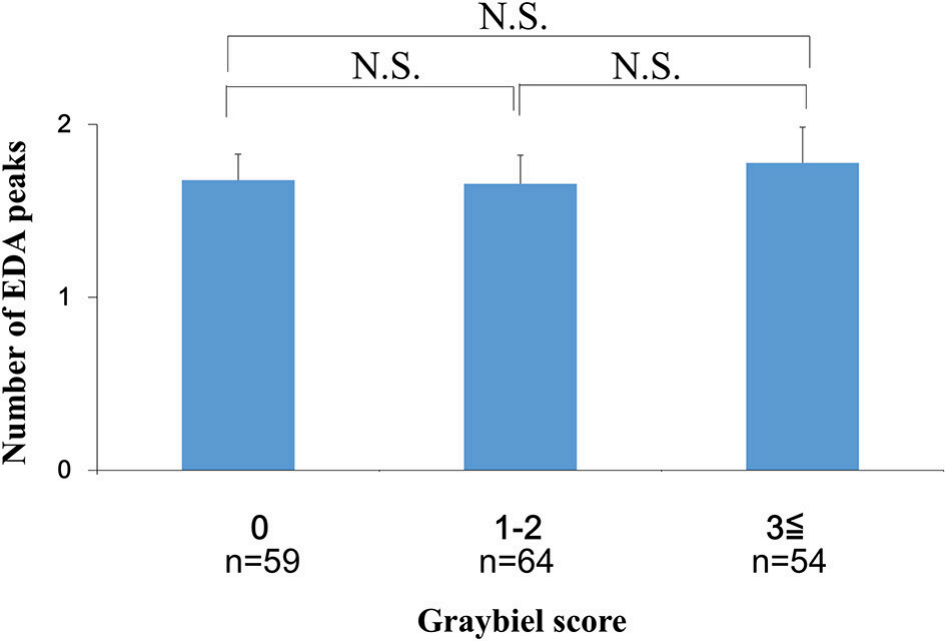
Evaluation of the number of EDA peaks and subjective autonomic symptoms during spatial disorientation training using the Q sensor and Graybiel criteria, respectively. We found no significant differences (*p* > 0.05) in the number of EDA peaks between the 0, 1–2, and ≥3 points groups. EDA, electrodermal activity. The error bars represent the mean (S.E.) in all graphs.

**Figure 7:**
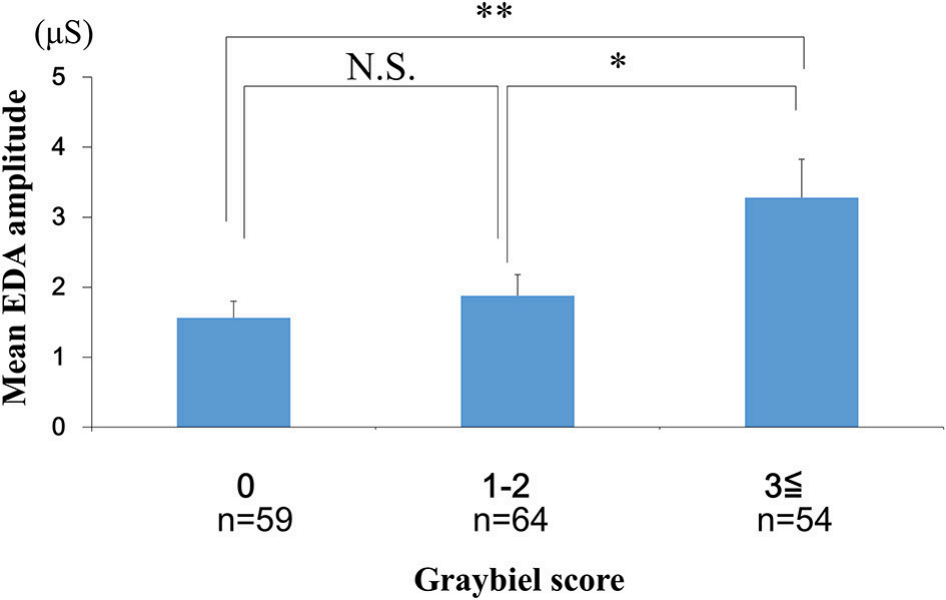
Evaluation of the mean EDA amplitude and subjective autonomic symptoms during spatial disorientation training using the Q sensor and Graybiel criteria, respectively. The mean amplitude was significantly increased in the ≥3 points group compared with the 0 points (*p* < 0.01) and the 1–2 points groups (*p* < 0.05). EDA, electrodermal activity. The error bars represent the mean (S.E.) in all graphs. ^*^p < 0.05, ^**^p < 0.01.

We investigated the number of EDA peaks, as well as the median and mean EDA amplitude in the 0 points, 1–2 points, and ≥3 points groups (Graybiel score). We found no significant changes in the number of EDA peaks during spatial disorientation training between the 0 points group, 1–2 points group, or ≥3 points group (Figure 6). The median EDA amplitudes were as follows: 0.80 μS (0 points group), 0.09 μS (1–2 points group), and 1.52 μS (≥3 points group). The mean EDA amplitude was significantly higher in the ≥3 points group compared with the 0 points group (*p* < 0.01) and the 1–2 points group (*p* < 0.05) (Figure 7). However, we observed no significant differences between the 0 points and 1–2 points groups (Figure 7).

### Evaluation of the Area Under the Curve for the EDA Data and Subjective Autonomic Symptoms

**Figure 8:**
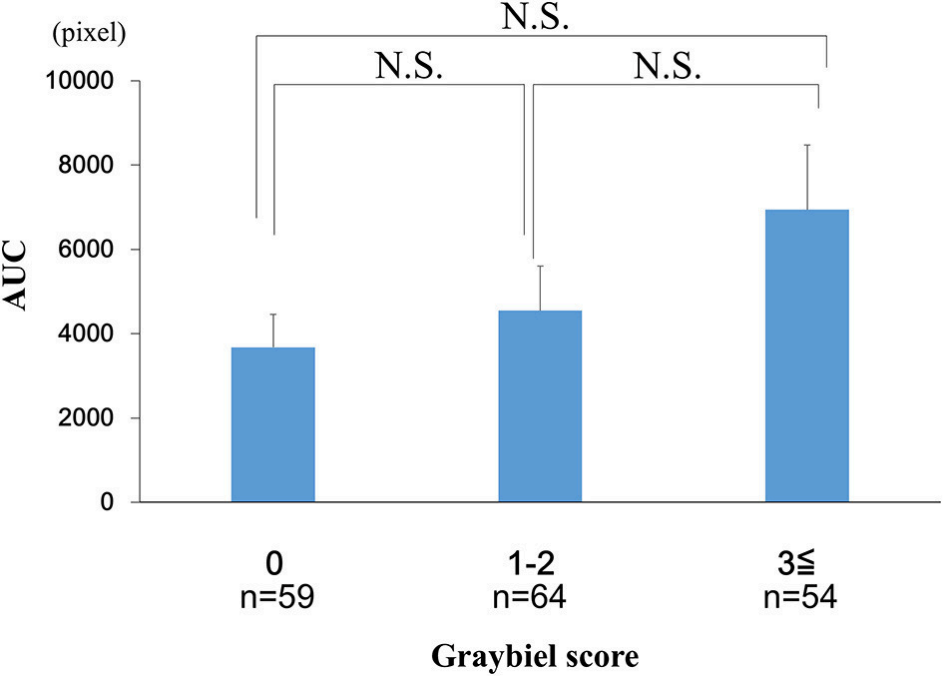
Evaluation of the AUC of the EDA data and subjective autonomic symptoms during spatial disorientation training using Q sensor and Graybiel criteria, respectively. We found no significant differences (*p* > 0.05) in the AUC between the 0, 1–2, and ≥3 points groups. AUC, area under the curve. EDA, electrodermal activity. The error bars represent the mean (S.E.) in all graphs.

We examined the AUC values for the EDA data. We divided the AUC data into three groups according to the Graybiel score (0, 1–2, and ≥3 points groups) and performed statistical analyses. The analyses revealed no significant differences between the 0 points group, 1–2 points group, and the ≥3 points group (Figure 8).

### Evaluation of EDA Data and Subjective Autonomic Symptoms for Each of the Seven Graybiel Diagnostic Categorizations

**Figure 9:**
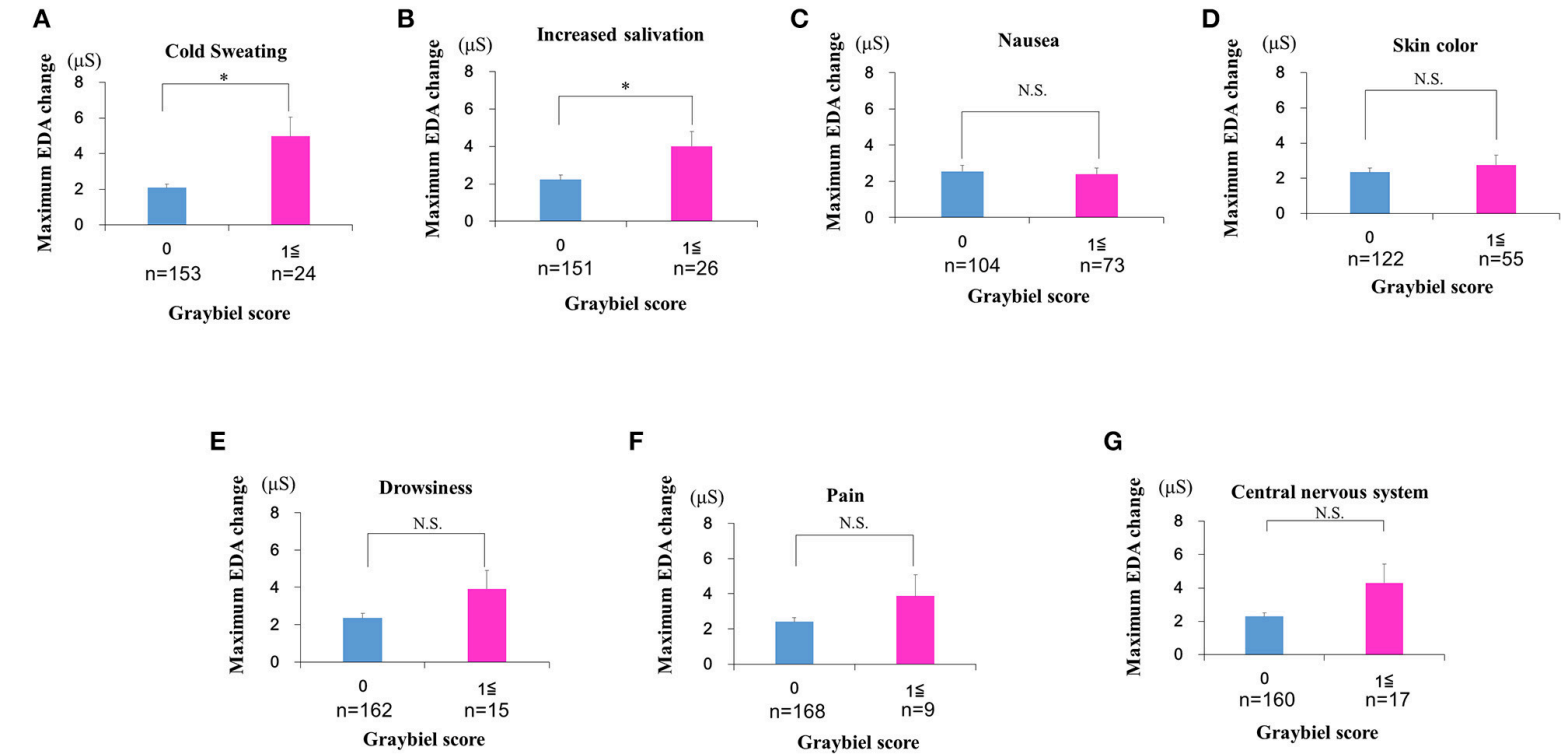
Evaluation of EDA using the Q sensor and subjective autonomic symptoms during spatial disorientation training for each of the seven Graybiel diagnostic categorizations. **(A–G)** The maximum amount of EDA change was significantly higher in the ≥1 points group compared with the 0 points group for “cold sweating” and “salivation” (*p* < 0.05, each) **(A,B)**. However, we observed no significant changes between the 0 points group and the ≥1 points group for the other five autonomic symptoms **(C–G)**. The error bars represent the mean (S.E.) in all graphs. ^*^p < 0.05.

Finally, we divided the participants into groups based on whether they had Graybiel scores of 0 or ≥1 for all seven Graybiel diagnostic criteria (nausea, skin color, cold sweating, increased salivation, drowsiness, pain, central nervous system) and evaluated changes in EDA. For cold sweating, the maximum EDA change was significantly higher in the ≥1 points group compared with the 0 points group (*p* < 0.05, Cohen's *d* = 1.70) (Figure 9A). For salivation, the maximum EDA change was also significantly higher in the ≥1 points group compared with the 0 points group (*p* < 0.05, Cohen's *d* = 1.02) (Figure 9B). Eight participants scored ≥1 point for both salivation and cold sweating symptoms.

However, we observed no significant differences between the 0 points and ≥1 points groups for the other five autonomic symptoms (Figures 9C–G). For nausea, the maximum EDA change was not significantly higher in the ≥1 points group compared with the 0 points group (*p* > 0.05, Cohen's *d* = 0.09) (Figure 9C). For skin color, the maximum EDA change was not significantly higher in the ≥1 points group compared with the 0 points group (*p* > 0.05, Cohen's *d* = 0.29) (Figure 9D). For drowsiness, the maximum EDA change was not significantly higher in the ≥1 points group compared with the 0 points group (*p* > 0.05, Cohen's *d* = 0.88) (Figure 9E). For pain, the maximum EDA change was not significantly higher in the ≥1 points group compared with the 0 points group (*p* > 0.05, Cohen's *d* = 0.82) (Figure 9F). For central nervous system symptoms, the maximum EDA change was not significantly higher in the ≥1 points group compared with the 0 points group (*p* > 0.05, Cohen's *d* = 1.14) (Figure 9G).

## Discussion

Spatial disorientation is a major risk for pilots (30, 31), and spatial disorientation-related accidents have been reported in many fighter aircraft and rotorcrafts (2, 32, 33). Therefore, in the interests of aviation safety, spatial disorientation training is considered to be important for pilots and pilot candidates (11–14). However, spatial disorientation affects the vestibular and visual systems independently and simultaneously. Thus, objective assessments of spatial disorientation and motion sickness can be difficult to conduct, as are scientific evaluations of the efficacy of spatial disorientation training. In addition, to the best of our knowledge, no previous studies have examined which types of spatial disorientation training are most effective for reducing spatial disorientation.

EDA reflects electrical changes measured at the skin surface that arise when the skin receives innervating signals from the brain. Previous experiments on spatial disorientation and motion sickness have used EDA as an indicator of changes in autonomic nervous system symptoms, revealing that EDA levels are sensitive to simulator sickness (14, 21).

One recent study reported that various physiological changes occurred during boarding in a flight simulator, and proposed that measuring EDA changes could provide a useful indicator (34). Although this previous study was similar to the current study, the details of the device used for measurement were not described, and it is possible that multiple electrodes were attached in the same way as in earlier studies.

With the recent development of wrist-worn EDA measuring devices, problems associated with attaching multiple electrodes and unexpected electrode detachment have been effectively resolved (18, 22). Accordingly, in the present study, we investigated the relationship between EDA changes and subjective autonomic symptoms using a novel wrist-worn EDA measurement device.

To evaluate subjective autonomic symptoms, we used the Graybiel diagnostic score (29) and quantified the severity of autonomic symptoms in our research. Graybiel diagnostic score has been established as a method for evaluating the severity of autonomic symptoms (29), and there have been many reports using this diagnostic score in the past (35–39). Recently, the Simulator Sickness Questionnaire (SSQ) has been used as a subjective evaluation method (40). The items regarding autonomic symptoms examined by the SSQ are relatively similar to Graybiel diagnosis. However, the SSQ is literally an evaluation of autonomic symptoms focusing on simulator sickness, which is different from the purpose of the current study. In addition, the calculation method for scaling in the SSQ is relatively complex. For these reasons, we used Graybiel diagnosis in the current study, which has been widely used for subjective evaluation in motion sickness research (29).

In the current study, few participants exhibited high Graybiel scores. Therefore, as shown in Figures 4, 6–8, we re-grouped participants so that the number was approximately equal between groups (*n* = 59, 64, 54). These groups corresponded to Graybiel cut-off values of 0, 1–2, and ≥3 (Figures 4, 6–8). In Figure 9, we further simplified the groupings, as there were seven items to consider. We separated participants into those who had no symptoms (0) and those who had some symptoms (≥1). Based on this classification, we examined the relationship between EDA and autonomic nervous system symptoms.

To date, the relationship between the severity of spatial disorientation and changes in EDA remains unclear. In one previous study, spatial disorientation training was performed using a simulator similar to that used in the present study, measuring several physiological markers and reporting that the change in EDA was not significantly different compared with that before training (22). However, it was reported that the EDA amplitude was higher during experimental conditions, although this increase did not reach statistical significance (22). However, it is possible that a significant difference was not found because the sample size used in the final analysis was reduced from 16 to 12 due to erroneous electrode placement in four of the participants (22).

In contrast to the previous study described above, in the current study we were able to measure EDA in a large number of participants. In addition, unlike past reports, we demonstrated a simple method for measuring EDA during spatial disorientation training with a limited timeframe using a compact wrist-worn device (18, 22). To our knowledge, this is the first study to report EDA measurement with a wrist-worn device, and to evaluate the correlation between autonomic symptoms and EDA during spatial disorientation training. Although the results revealed a relationship between autonomic symptoms and the degree of EDA change and mean amplitude, this was limited to instances in which the participants experienced strong symptoms, and two kinds of autonomic symptoms.

In the case of spatial disorientation and motion sickness, the role of sympathetic and parasympathetic nervous function has not yet been clarified. Furthermore, it remains unclear whether dysfunction of the autonomic nervous system causes spatial disorientation and motion sickness, or whether spatial disorientation and motion sickness cause dysfunction of the autonomic nervous system. Gordon et al. examined the components of saliva as an objective assessment of motion sickness, reporting that the amount of amylase in saliva was significantly higher in people who were prone to motion sickness compared with that in people who were not, suggesting that salivary amylase levels could provide an indicator of susceptibility to motion sickness (26). Because amylase secretion in saliva is regulated by the sympathetic nervous system, the authors concluded that the sympathetic nervous system may work more actively in people who are prone to motion sickness.

In addition, one previous study measured heart rate variability (HRV) to evaluate the state of spatial disorientation and motion sickness by capturing changes in the autonomic nervous system (41). The power spectra of HRV contain high frequency (HF) components indicating parasympathetic nervous activity, low frequency (LF) components reflecting both sympathetic and parasympathetic activity and relating to the average heart rate of the subject, and LF/HF components reflecting the balance between sympathetic nervous system activity and parasympathetic nervous system activity (41, 42). The study mentioned above indicated that sympathetic nervous function (such as decreased HF and increased LF/HF) became active as symptoms of motion sickness appeared and the condition became severe (41).

A previous experiment, in which motion sickness was caused by loading a visual stimulus stepwise, reported that HF decreased first, followed by an increase in LF/HF (43). Thus, the autonomic nervous system activity at the onset of motion sickness was thought to be caused by suppression of parasympathetic nervous function rather than promotion of sympathetic nervous function.

Despite the different indicator items, these reports indicate that hyperactivity of the sympathetic nervous system could provide an indicator of susceptibility of spatial disorientation and motion sickness. This finding suggests that spatial disorientation and motion sickness may cause suppression of parasympathetic nervous function and activation of sympathetic nervous function, indicating autonomic nervous system dysfunction. Based on these reports, we considered that inter-individual autonomic symptom variability was caused by differences in the susceptibility of participants to spatial disorientation. In the current experiments, all participants were healthy pilot candidates of the Japan Air Self-Defense Force who did not have any abnormal physical conditions. The results revealed that the symptoms of cold sweating and salivation increased with increasing EDA. However, depending on physical conditions and daily fluctuations of autonomic nervous activity, small inter-individual differences may exist, even if the same experiment is performed. Further verification will be necessary to confirm whether autonomic nervous dysfunction is the cause or the result of spatial disorientation and motion sickness, and to investigate how much EDA and autonomic symptoms are affected by physical condition.

When we examined participants with total Graybiel scores of ≥3 points, we observed no significant (*p* > 0.05) correlation (*r* = −0.059) between the maximum EDA change and total Graybiel score (Figure 3). In addition, we found no significant (*p* > 0.05) differences between the AUC of the EDA data and the total Graybiel score (Figure 6). We speculate that this finding was due to a combination of individual differences between participants and several important experimental limitations, as described below.

In this study, EDA measurements were carried out during 30 min of spatial disorientation training. For example, although there was no significant difference between each group in the AUC of the EDA results, significant differences were observed among some groups in maximum EDA change and mean amplitude. We speculate that the correlation between maximum EDA change, mean amplitude and Graybiel scores could be explained by the maximum EDA change representing temporary extreme motion sickness, with mean amplitude representing a sustained level in the measurement period of motion sickness. Thus, we consider that maximum EDA change and mean amplitude are appropriate parameters for examining EDA changes.

EDA increased in all subjects immediately after the Coriolis illusion was started. The Coriolis illusion has been used to investigate various physiological changes in motion sickness and spatial disorientation (44). Although the current study only examined changes in EDA, the Coriolis illusion is also considered to be a suitable method for investigating physiological changes in spatial disorientation.

The present findings should be interpreted within the context of the following limitations. First, we analyzed the EDA data without considering individual differences in EDA values under normal circumstances. Importantly, we investigated the maximum change in EDA values from immediately before the start of the spatial disorientation training to the end of training in all participants. The skin conductance level (SCL) is a numerical value reflecting tonic EDA (43). However, the Q sensor used in this study was unable to easily measure SCL. Although participants' original tonic EDA appeared to be slightly larger than 0 microsiemens (μS), a value of 0 μS was considered to indicate tonic EDA because of the limitations of the device. We considered the value of EDA just before spatial disorientation training as the tonic state, and considered the change from the tonic state to be the phasic state. However, when we reviewed the measured EDA data, there were no participants with high EDA values related to excitement and anxiety before training, and the EDA value immediately before the start of the training in all subjects was 0 μS. Therefore, we assumed 0 μS as the reference value in all cases, and calculated the EDA change amount subsequently.

Second, we did not consider the impact of filtering during EDA measurement. The Q sensor we used initially had no filter. However, using a filter introduces the possibility of distorting the EDA signal without artifacts. Importantly, no artifacts were detected in the current study. Thus, we consider that filtering was unlikely to have substantially influenced our results. Nevertheless, the potential impact of filters on EDA measurement should be considered in future studies.

Third, when participants flexed their necks to experience the Coriolis illusion, there may have been a difference in neck bending speed (21). Therefore, the strength of the Coriolis illusion may have differed between participants, potentially affecting the subsequent changes in autonomic nervous system symptoms and EDA.

Fourth, participants may have under-declared or over-reported subjective symptoms. This is a common limitation of subjective evaluation methods (24, 25), and highlights the importance of objective evaluation methods, such as the wrist-worn EDA device.

Fifth, all participants were pilot candidates in the Japan Air Self-Defense Force, which is predominantly male. The gender imbalance in our sample may have affected the results.

Sixth, all participants wore the Q sensor on their left wrists. The left hand of each participant was in a fixed position beside the seat in the spatial disorientation device. Interestingly, a previous study reported that human EDA exhibits left-right asymmetry (45). Because of the tight training schedule of the candidate pilots, we were not able to verify EDA asymmetry in the participants by measuring it on both sides. We hope to address this issue in future studies. With respect to the issue of movement-based changes in EDA, participants in our study were unable to move their left hands during the training session. Although we did not find evidence of pronounced movement-based noise, low-amplitude noise accompanying the movement of the spatial disorientation training device was present. However, this low-amplitude noise did not appear to affect the EDA data. Therefore, we believe that the results of this experiment accurately reflected the severity of spatial disorientation and motion sickness.

Seventh, we evaluated the sympathetic component of the autonomic nervous system, but were unable to evaluate the parasympathetic nervous system in this experiment. Previous studies indicated that sympathetic activity was increased as symptoms of motion sickness appeared and as its severity increased (41, 46). However, the precise roles of the sympathetic and parasympathetic nervous systems at the time of onset of spatial disorientation and motion sickness are not well-understood. In the current experiment, we evaluated the autonomic nervous system and focused on EDA. However, we only evaluated the sympathetic nervous system, and were unable to evaluate the parasympathetic system because the interior of the spatial disorientation training device we used in this experiment was narrow, making it difficult to use a large biological monitoring system to measure both the sympathetic and parasympathetic nervous systems. In the future, we plan to evaluate the parasympathetic nervous system in addition to the sympathetic nervous system.

## Conclusion

Despite the limitations described above, the current results, taken together with the findings of previous studies, indicate that EDA can be easily measured using a compact device, and that autonomic nervous system activity can be functionally evaluated during spatial disorientation training. Our findings also indicate that the severity of spatial disorientation and motion sickness can be evaluated using this method. Future research will extend our understanding of EDA and autonomic symptoms with respect to spatial disorientation and motion sickness.

## Author Contributions

AT organized the whole study. AT, TI, HO, YT, and YW were involved in the study concept and design. AT, TI, HO, and YW performed the research and analyzed the data. MK and YT provided laboratory facility support. AT wrote the manuscript. All authors reviewed the manuscript.

### Conflict of Interest Statement

The authors declare that the research was conducted in the absence of any commercial or financial relationships that could be construed as a potential conflict of interest.
